# SlMYC2‐SlMYB12 module orchestrates a hierarchical transcriptional cascade that regulates fruit flavonoid metabolism in tomato

**DOI:** 10.1111/pbi.14510

**Published:** 2024-11-07

**Authors:** Heng Deng, Mengbo Wu, Yi Wu, Xiangxia Xiao, Zhuo Gao, Huirong Li, Nan Hu, Yongfeng Gao, Don Grierson, Mingchun Liu

**Affiliations:** ^1^ Key Laboratory of Bio‐Resource and Eco‐Environment of Ministry of Education, College of Life Sciences Sichuan University Chengdu China; ^2^ School of Life Science and Engineering Southwest University of Science and Technology Mianyang China; ^3^ College of Chemistry, Biology and Environment Yuxi Normal University Yuxi China; ^4^ College of Biology and Food Engineering Anyang Institute of Technology Anyang China; ^5^ School of Biosciences University of Nottingham Loughborough UK

**Keywords:** tomato, flavonoids, MYC2, transcriptional regulation

Flavonoids are a class of secondary metabolites widely present in plants that serve various functions, such as pigmentation, UV protection and defence against pathogens and herbivores (Naik *et al*., 2022). Numerous structural genes and transcription factors involved in flavonoid biosynthesis have been successfully identified (Naik *et al*., 2022), which has significantly enhanced our understanding of the molecular mechanisms underlying flavonoid production in plants.

MBW (MYB–bHLH–WDR) transcription factor protein complexes are crucial regulators of flavonoid biosynthesis (Xu *et al*., 2015). Among these, MYB transcription factors have been extensively studied as major regulators of the MBW complex, modulating flavonoid production in various plants (Xu *et al*., 2015). In tomato, SlMYB12 also plays a crucial role in the accumulation of flavonoids in the fruit by positively regulating flavonoid biosynthesis genes, such as *CHS*, *CHI*, *F3H* and *FLS1* (Zhang *et al*., 2015).

MYC2, a basic helix–loop–helix (bHLH) transcription factor, is crucial in the jasmonic acid (JA) signalling pathway (Liu *et al*., 2019). At low JA‐Ile levels, JAZ proteins recruit the co‐repressor TOPLESS, preventing MYC2 from activating downstream genes. With increased JA‐Ile levels, JAZ binds to COI1, leading to its degradation mediated by the SCF^COI1^ ubiquitin ligase complex (Liu *et al*., 2019). Subsequently, MED25 interacts with free MYC2, recruiting the histone acetylase HAC1, which regulates the acetylation level of Lys‐9 of histone H3 in the promoter regions of MYC2 target genes, thereby activating their expression (Liu *et al*., 2019). In tomato fruits, SlMYC2 has been reported to positively regulate flavonoid content (Zhang *et al*., 2022); however, the underlying mechanisms remain unclear. We found that *SlMYC2* displayed relatively high expression during the late ripening stages (from breaker (Br) stages Br + 7 to Br + 15) (Figure S1a), indicating its involvement in ripening. A subcellular localization assay showed that SlMYC2‐GFP localized to the nucleus (Figure S1b), suggesting that it functions as a transcription factor. To investigate the functional significance of SlMYC2 in ripening, we generated two *SlMYC2* knockout (*KO*) lines using CRISPR/Cas9 with one sgRNA (Figure 1a). Key structural genes involved in flavonoid biosynthesis, including *SlCHS1*, *SlCHS2*, *SlF3H*, *SlF3′H* and *SlFLS*, along with the transcription factor *SlMYB12*, were significantly downregulated in Br + 7 fruits of *SlMYC2‐KO* lines (Figure 1b, Figures S2 and S3). Moreover, the levels of flavonoids, including naringenin, rutin, eriodictyol, nicotiflorin and caffeic acid, as well as that of the flavonoid derivative chlorogenic acid were significantly lower in *SlMYC2* *‐KO* fruits than in WT fruits (Figure 1c), suggesting a positive regulatory role of MYC2 in flavonoid accumulation in tomato fruits. Despite changes in flavonoid content, ripening onset, fruit firmness and carotenoid levels in *SlMYC2‐KO* fruits remained similar to those in WT fruits (Figure S4), indicating that SlMYC2 specifically activates flavonoid biosynthesis without affecting broader ripening processes.

**Figure 1:**
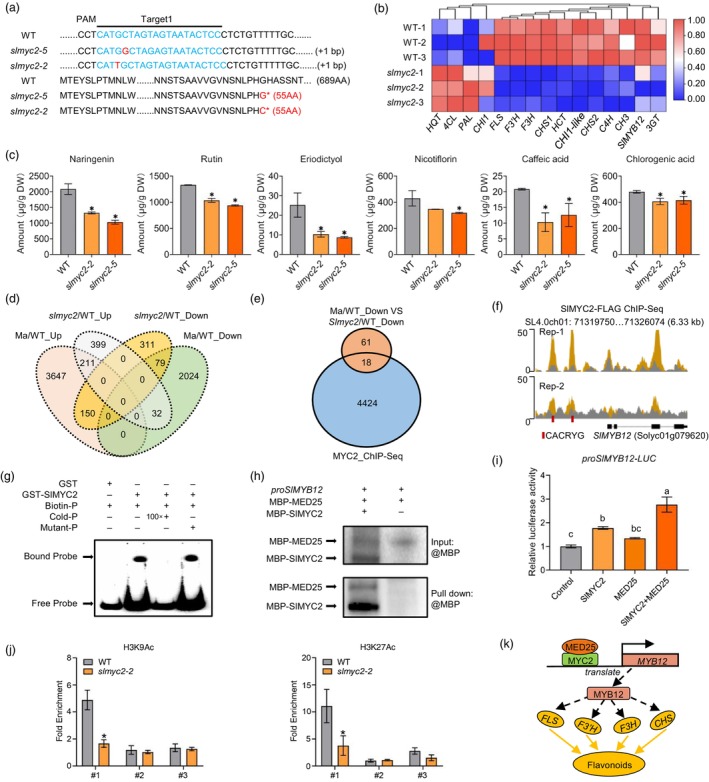
SlMYC2‐MED25 complex activates the transcription of *SlMYB12* to increase flavonoid accumulation in tomato fruits. (Details relevant to Figure 1a–k are provided in Supporting Information Figure legend).

The activation of target genes by MYC2 relies on its interaction with the mediator subunit MED25, which promotes the acetylation of Lys‐9 of histone H3 (H3K9Ac) in downstream gene promoter regions (Breeze, 2019). To explore the molecular mechanism by which SlMYC2 promotes flavonoid accumulation in fruits, we conducted a combined analysis of differentially expressed genes (DEGs) between the WT and *SlMYC2‐KO* lines (Data sets S1 and S2), as well as the previously reported DEGs between the WT and *MED25*‐AS (Ma) lines (Deng *et al*., 2023). The results revealed 79 genes that were simultaneously downregulated in both the *SlMYC2‐KO* and Ma lines (Figure 1d, Data Set S3), suggesting that these genes might be positively regulated by the SlMYC2‐MED25 complex.

To identify the direct targets of the SlMYC2‐MED25 complex involved in flavonoid metabolism regulation, we performed a comprehensive comparison of 4442 putative SlMYC2 target genes previously identified through ChIP‐Seq analysis (Du *et al*., 2017) and the 79 genes found to be positively regulated by the SlMYC2‐MED25 complex in the present study. This integrated analysis identified a subset of 18 genes that were putative direct transcriptional targets activated by SlMYC2 (Figure 1e; Table S1). Notably, *SlMYB12* (*Solyc02g077790*), a key positive transcription factor in the tomato flavonoid pathway (Zhang *et al*., 2015), was among the 18 genes directly regulated by SlMYC2 (Table S1). Moreover, ChIP‐Seq data (Du *et al*., 2017) and EMSA confirmed the direct binding of SlMYC2 to the *SlMYB12* promoter at the CACRYG sites *in vivo* and *in vitro* (Figure 1f,g).

To further illustrate the regulatory role of the SlMYC2‐MED25 complex in *SlMYB12* expression, we first performed yeast two‐hybrid and split‐luciferase complementation assays and verified the interaction between SlMYC2 and MED25 both *in vitro* and *in vivo* (Figure S5). DNA pull‐down assays conducted using a biotin‐labelled *SlMYB12* promoter showed that the recruitment of MED25 to the *SlMYB12* promoter was dependent on SlMYC2 (Figure 1h). Moreover, transactivation assays demonstrated that the co‐expression of *SlMYC2* and *MED25* with the *proSlMYB12‐*LUC reporter in *Nicotiana benthamiana* leaf protoplasts resulted in a significant increase in the transcriptional activity of the *SlMYB12* promoter compared to the expression of *SlMYC2* or *MED25* alone (Figure 1i), further illustrating the role of the SlMYC2‐MED25 complex in transcriptional activation in *SlMYB12*. These data indicated that the SlMYC2‐MED25 complex influences flavonoid accumulation in tomato fruits by directly regulating *SlMYB12* expression.

The SlMYC2‐MED25 complex binds to the promoter regions of target genes by recruiting histone acetyltransferase HAC1, which increases histone H3 acetylation in the promoter regions, leading to chromatin relaxation and activation of target gene expression (Liu *et al*., 2019). We investigated the enrichment of two histone H3 modification markers, H3K9Ac and H3K27Ac, in the *SlMYB12* promoter region of both WT and *SlMYC2‐KO* fruits. Decreased levels of both H3K9Ac and H3K27Ac were found in *SlMYC2‐KO* fruits compared to those in WT fruits (Figure 1j), suggesting that the SlMYC2‐MED25 complex activates the expression of *SlMYB12* by modulating histone acetylation levels within the promoter region.

In conclusion, by combining analysis of the transcriptomes of *slmyc2* and *MED25*‐AS (Ma) lines with ChIP‐Seq data of SlMYC2, we identified the key transcription factor SlMYB12 as a direct target of the SlMYC2‐MED25 complex in regulating flavonoid metabolism (Figure 1k). Our study elucidates the molecular mechanism by which SlMYC2 regulates flavonoid metabolism in tomato fruits, thereby extending our understanding of the functional significance of SlMYC2 in fruit quality regulation.

## Conflict of interests

The authors declare no competing interests.

## Author contributions

M.L. and H.D. planned and designed the research; H.D., M.W., Y.W., X.X. and Z.G., performed experiments. H.L., N.H. and Y.G. analysed data. M.L. and H.D. wrote the manuscript and D.G. helped improve the manuscript.

## Supporting information


**Figure S1** The expression levels of *SlMYC2* vary across different tissues and subcellular localization analysis of SlMYC2.
**Figure S2** The expression levels of *CHS1*, *CHS2*, *HCT*, *CH3*, *F3H*, *F3′H*, *FLS* and *MYB12* were obtained from the qPCR data.
**Figure S3** RNA‐seq analysis of WT and *SlMYC2‐KO* fruits.
**Figure S4** SlMYC2 does not affect tomato fruit ripening process and carotenoid accumulation.
**Figure S5** Verification of interaction between SlMYC2 and MED25.
**Table S1** Putative transcriptional targets of the SlMYC2–SlMED25 complex by combining RNA‐seq and ChIP‐seq data.
**Data Set S1** Differentially expressed genes (DEGs) between *slmyc2* and WT fruits.
**Data Set S2** Gene Expression (TPM) in *slmyc2* and WT Fruits.
**Data Set S3** The gene locus numbers in the venn diagram of Figure S3a.
**Data Set S4** The kyoto encyclopedia of genes and genomes (KEGG) analysis of DEGs between *slmyc2* and WT fruits.
**Data Set S5** Gene expression levels (TPMs) in the heat maps of Figure 1b and Figure S4e.
**Data Set S6** List of primers used in this study.
**Data Set S7** Flavonoid content and reference standard detection.
**Data Set S8** Carotenoid content and reference standard detection.
**Data Set S9** Statistical analysis.

## Data Availability

The RNA‐Seq data of this study are openly available at the National Genomics Data Center (Beijing Institute of Genomics, Chinese Academy of Sciences) at https://ngdc.cncb.ac.cn/gsa/ (reference number: CRA015972). Published RNA‐Seq and ChIP‐Seq data are deposited in the National Genomics Data Center under accession numbers CRA003758 and CRA000222, respectively.
