# Supplementary material for: SlMYC2‐SlMYB12 module orchestrates a hierarchical transcriptional cascade that regulates fruit flavonoid metabolism in tomato
Source: Plant Biotechnol J. 2024 Nov 7;23(2):477–9. doi: 10.1111/pbi.14510 (PMC11772319; doi:10.1111/pbi.14510)
Supplement: Supplementary file 1 — Figure S1 The expression levels of SlMYC2 vary across different tissues and subcellular localization analysis of SlMYC2. Figure S2 The expression levels of CHS1, CHS2, HCT, CH3, F3H, F3′H, FLS and MYB12 were obtained from the qPCR data. Figure S3 RNA‐seq analysis of WT and SlMYC2‐KO fruits. Figure S4 SlMYC2 does not affect tomato fruit ripening process and carotenoid accumulation. Figure S5 Verification of interaction between SlMYC2 and MED25. Table S1 Putative transcriptional targets of the SlMYC2–SlMED25 complex by combining RNA‐seq and ChIP‐seq data. Data Set S1 Differentially expressed genes (DEGs) between slmyc2 and WT fruits. Data Set S2 Gene Expression (TPM) in slmyc2 and WT Fruits. Data Set S3 The gene locus numbers in the venn diagram of Figure S3a. Data Set S4 The kyoto encyclopedia of genes and genomes (KEGG) analysis of DEGs between slmyc2 and WT fruits. Data Set S5 Gene expression levels (TPMs) in the heat maps of Figure 1b and Figure S4e. Data Set S6 List of primers used in this study. Data Set S7 Flavonoid content and reference standard detection. Data Set S8 Carotenoid content and reference standard detection. Data Set S9 Statistical analysis. [file PBI-23-477-s001.zip › pbi14510-sup-0002-supinfo.docx]

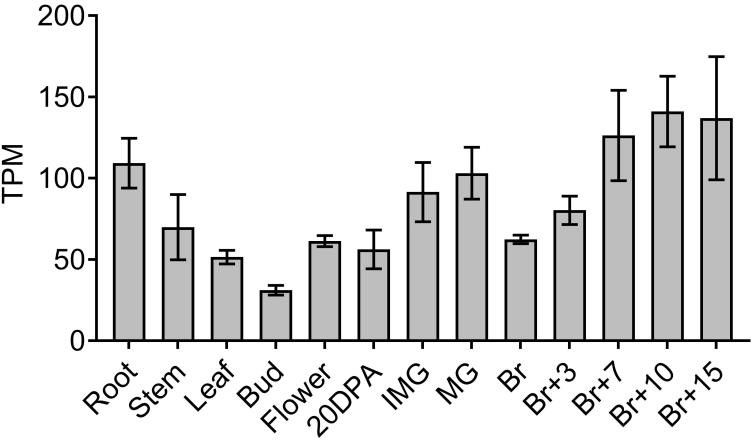
(a) (b)

GFP BF Merge


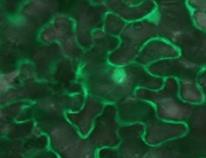

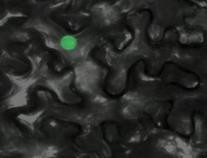

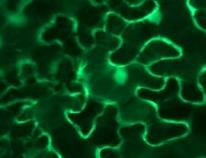

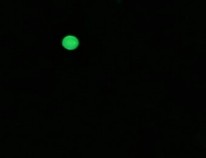

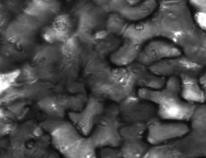

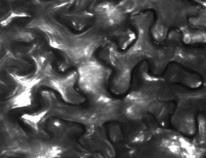


SlMYC2-eGFP

Freee GFP

Figure S1 The expression levels of *SlMYC2* vary across different tissues and subcellular localization analysis of SlMYC2. (a) The expression levels of *SlMYC2* vary across different tissues and stages of fruit development and ripening. Data are shown as means ± standard deviation (SD) (n = 3). 20DPA, 20 days post anthesis; IMG, immature green; MG, immature green; Br, breaker; Br+3-15, 3-15 days after the Br stage. (b) Subcellular localization analysis of SlMYC2. Green fluorescent protein (GFP)-tagged fusion proteins were expressed in tobacco leaves following transfection under the control of the 35S promoter.


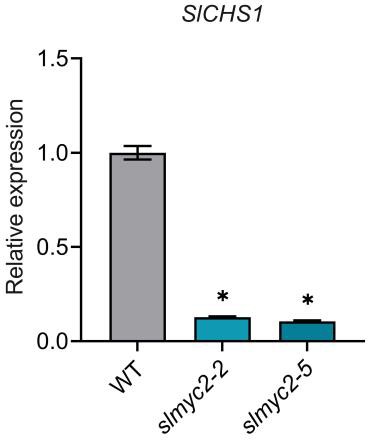

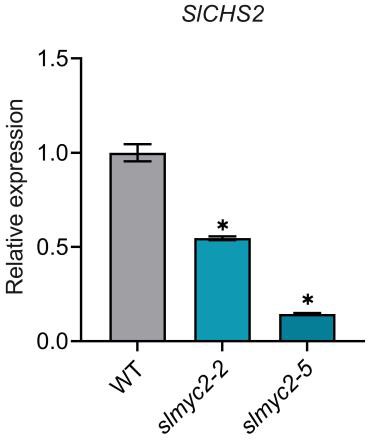

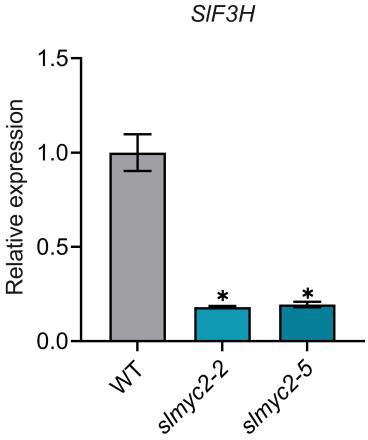

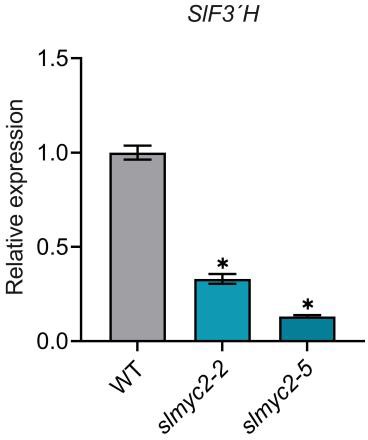

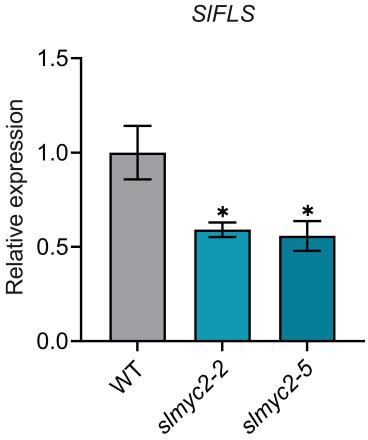

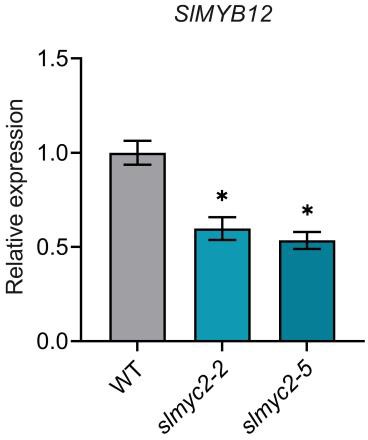


Figure S2 The expression levels of *CHS1*, *CHS2*, *HCT*, *CH3*, *F3H*, *F3’H*, *FLS* and *MYB12* were obtained from the qPCR data. Relative mRNA levels of WT were normalized to 1, *Slactin* were used as an internal control. Data are shown as means ± SD from six biological replicates. Asterisks indicate statistical significance using Student’s *t*-test, P＜0.05.

(a)


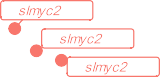


(b)

(c)


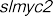

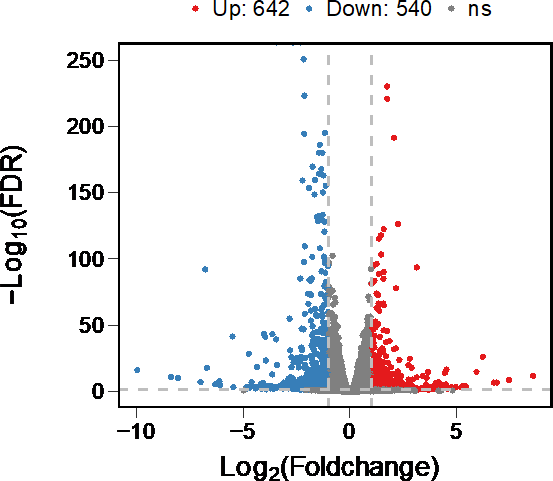


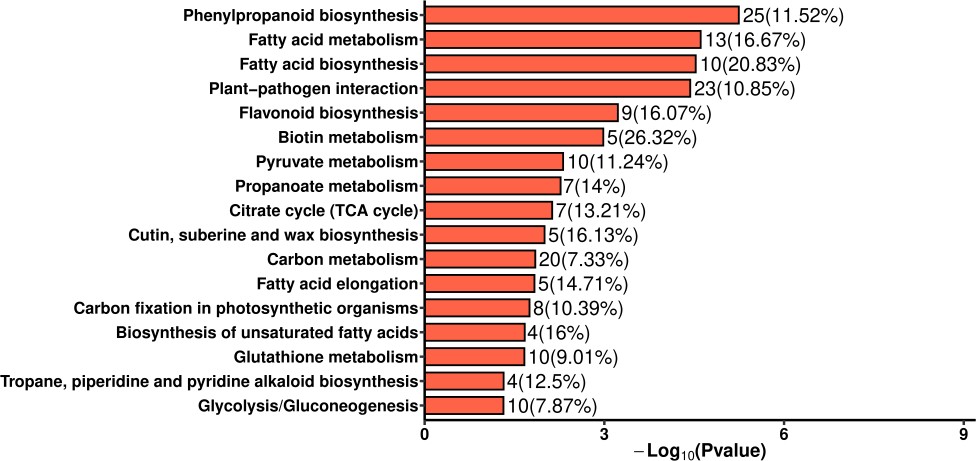


Figure S3 RNA-seq analysis of WT and *SlMYC2-KO* fruits. (a) Principal component analysis (PCA) of RNA-seq dataset. The samples were collected from WT and *slmyc2* fruits at the Br+5 stage, with three biological replicates for each genotype. (b) Volcano diagrams of DEGs in WT and *slmyc2* fruits at 5 DPI. (c) KEGG analysis of 1182 DEGs in WT and *slmyc2* fruits.

(a)

DPA 41 42 43 44 45 46 47 48 49 50

WT


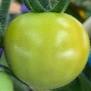

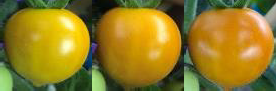

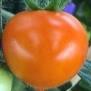

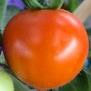

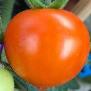

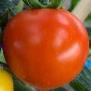

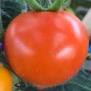

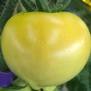

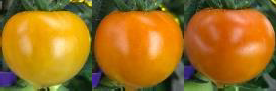

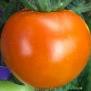

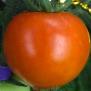

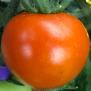

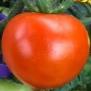

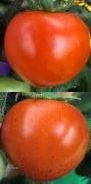

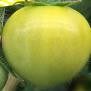

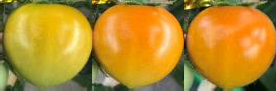

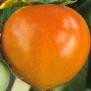

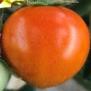

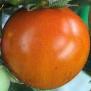

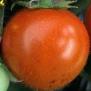

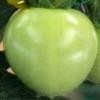

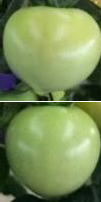


(d) (e)


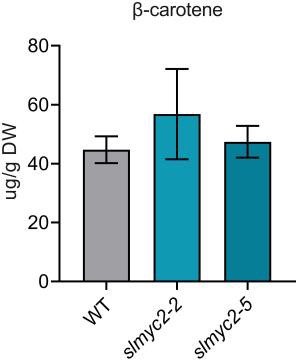

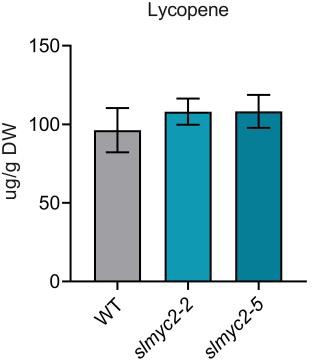

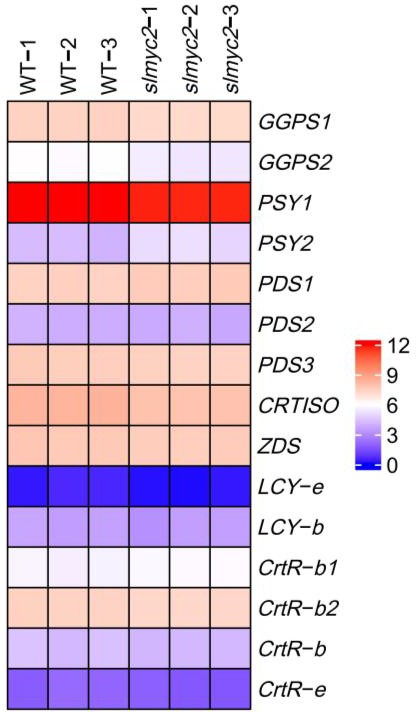
*slmyc2-2 slmyc2-5*


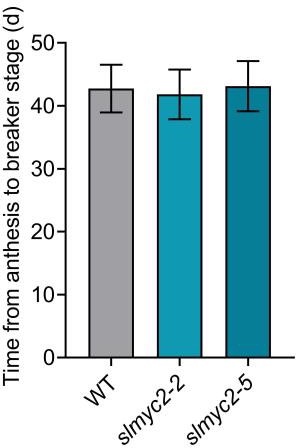
(b)

(c)


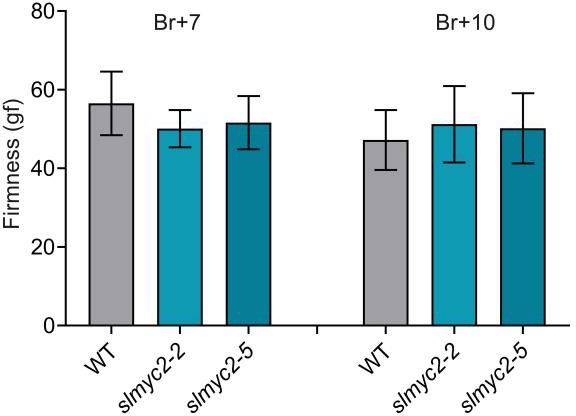

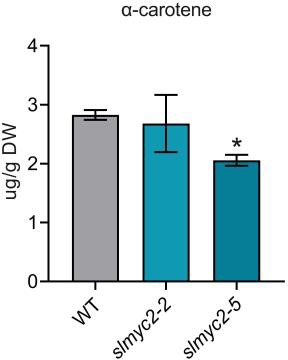

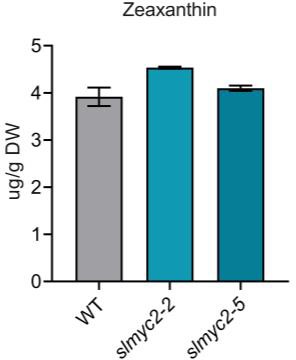

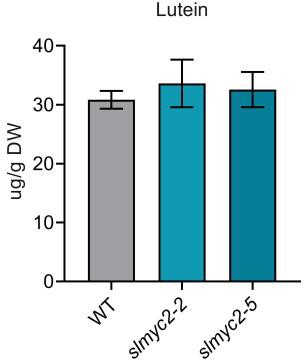


Figure S4 SlMYC2 does not affect tomato fruit ripening process and carotenoid accumulation. (a) Different

ripening stages in *slmyc2* and WT lines. (b) Time from anthesis to the Br stage in *slmyc2* and WT lines. Data are presented as means ± standard deviation (SD) (n = 15). (c) Fruit firmness of *slmyc2* and WT fruits at the Br+7 and Br+10 stages. Data are presented as means ± standard deviation (SD) (n = 15). (d) Carotenoid content in *slmyc2* and WT lines. Data are presented as means ± SD from six biological replicates. Asterisks indicate statistical

significance using Student’s t-test, P < 0.05. (e) Expression of carotenoid biosynthesis genes in *slmyc2* and WT lines. Data are presented as means ± SD from three biological replicates. Asterisks indicate statistical significance using Student’s *t*-test, P < 0.05.

(a)

pGADT7 pGBKT7

pGADT7 pGBKT7-MED25

pGADT7-MYC2 pGBKT7

pGADT7-MYC2 pGBKT7-MED25


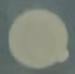
pGADT7-T pGBKT7-P53

SD-LW


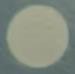


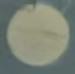


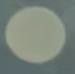


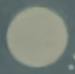


SD-LWAH


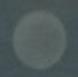

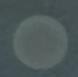

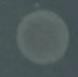

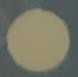


(b)


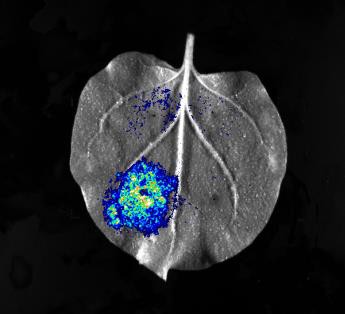

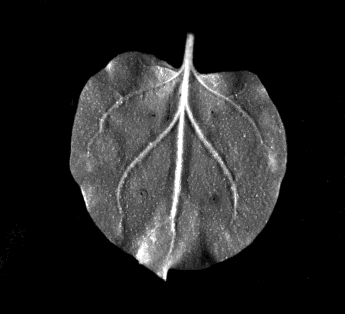


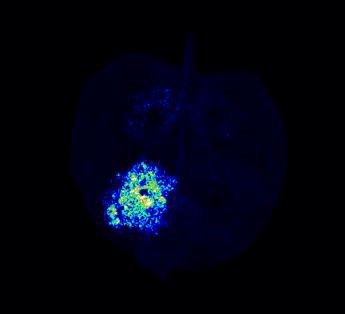

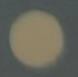
Figure S5 Verification of interaction between SlMYC2 and MED25. (a) Y2H Assay of Interaction Between MYC2 and MED25. pGBKT7-MED25 was used as bait and pGADT7-MYC2 as prey. Negative controls included co-transformations of empty-pGBKT7 with empty-pGADT7, empty-pGBKT7 with pGADT7-MYC2, and pGBKT7-MED25 with empty-pGADT7. pGADT7-T co-transformed with pGBKT7-P53 served as the positive control. (b) Split-Luciferase Complementation Assay of interaction between MYC2 and MED25. MYC2-nLUC fusion constructs were co-transformed with cLUC-MED25 as the experimental condition. Negative controls included co-transformations of MYC2-nLUC with cLUC-MBP or cLUC, and nLUC with cLUC.

MYC2-nLUC MYC2-nLUC cLUC-MBP cLUC

MYC2-nLUC nLUC cLUC-MED25 cLUC
