# Supplementary material for: SlMYC2‐SlMYB12 module orchestrates a hierarchical transcriptional cascade that regulates fruit flavonoid metabolism in tomato
Source: Plant Biotechnol J. 2024 Nov 7;23(2):477–9. doi: 10.1111/pbi.14510 (PMC11772319; doi:10.1111/pbi.14510)
Supplement: Supplementary file 1 — Figure S1 The expression levels of SlMYC2 vary across different tissues and subcellular localization analysis of SlMYC2. Figure S2 The expression levels of CHS1, CHS2, HCT, CH3, F3H, F3′H, FLS and MYB12 were obtained from the qPCR data. Figure S3 RNA‐seq analysis of WT and SlMYC2‐KO fruits. Figure S4 SlMYC2 does not affect tomato fruit ripening process and carotenoid accumulation. Figure S5 Verification of interaction between SlMYC2 and MED25. Table S1 Putative transcriptional targets of the SlMYC2–SlMED25 complex by combining RNA‐seq and ChIP‐seq data. Data Set S1 Differentially expressed genes (DEGs) between slmyc2 and WT fruits. Data Set S2 Gene Expression (TPM) in slmyc2 and WT Fruits. Data Set S3 The gene locus numbers in the venn diagram of Figure S3a. Data Set S4 The kyoto encyclopedia of genes and genomes (KEGG) analysis of DEGs between slmyc2 and WT fruits. Data Set S5 Gene expression levels (TPMs) in the heat maps of Figure 1b and Figure S4e. Data Set S6 List of primers used in this study. Data Set S7 Flavonoid content and reference standard detection. Data Set S8 Carotenoid content and reference standard detection. Data Set S9 Statistical analysis. [file PBI-23-477-s001.zip › pbi14510-sup-0003-supinfo.docx]

**Supplementary Figure legend**

**Figure legend**

**Figure 1** SlMYC2-MED25 complex activates the transcription of *SlMYB12* to increase flavonoid accumulation in tomato fruits. (a) Genotype of *SlMYC2-KO* lines; *slmyc2-5* and *slmyc2-7* are two independent mutants created using CRISPR/Cas9 genome editing. The protospacer adjacent motif is underlined. (b) Expression heatmap of flavonoid biosynthesis genes in WT and *sl**myc2.* The expression levels of *PAL*, *C4H*, *CHS1*, *CHS2*, *HCT*, *CH3*, *4CL*, *HQT*, *CHI1*, *CHI1-like*, *F3H*, *F3′H*, *FLS*, and *3GT* were obtained from the RNA-Seq data. (c) The levels of flavonoids and flavonoid derivative decreased in the *slmyc2* mutants. The levels of naringenin, eriodictyol, nicotiflorin, chlorogenic acid, caffeic acid, and rutin were measured using liquid chromatography-mass spectrometry (LC-MS) analysis. Data are shown as means ± standard deviation (SD) (n = 3). Asterisks indicate statistical significance using the Student’s *t*-test, *p <* 0.05. (d) Venn diagram illustrates the overlap between SlMYC2- and MED25-activated genes. Seventy-nine genes found in the overlapping region of *slmyc2*/WT_down and ma/WT_down are identified as co-activated genes of SlMYC2 and MED25 in ripening. *slmyc2* /WT_down, genes downregulated in the *slmyc2* mutant compared to WT; *slmyc2* /WT_up, genes upregulated in the *slmyc2* mutant compared to WT; Ma/WT_down, genes downregulated in the *med25*-RNAi line compared to WT; Ma/WT_up, genes upregulated in the *med25*-RNAi line compared to WT. (e) Venn diagram illustrates the intersection between the co-activated genes of SlMYC2 and MED25, and the target genes of SlMYC2. (f) Binding of SlMYC2 to the *SlMYB12* promoter regions shown by chromatin immunoprecipitation followed by sequencing. The orange and gray sequencing peaks represent two biological replicates and the inputs, respectively. The structure of *SlMYB12* is presented below, with black bars representing exons and lines representing introns. The direction of transcription is indicated by arrows in the lines. The red marks indicate the SlMYC2-FLAG binding site, CACRYG. (g) EMSA showed SlMYC2 binding to the CACAGG site of *SlMYB12* promoters. WT probe sequences containing the CACAGG site were biotin labeled. Competition for SlMYC2 binding was performed with 100× cold probe containing the CACAGG site or mutated control (AAAAAA). The symbols + and – represent presence and absence, respectively. (h) MED25 binds to the *SlMYB12* promoter through SlMYC2. Recombinant MBP-MED25 was incubated with a biotin-labeled *SlMYB12* promoter DNA fragment with or without MBP-MYC2 and then pulled down using streptavidin agarose beads. Immunoblots were probed with an anti-MBP antibody. (i) Transcriptional activation assay of *SlMYB12* promoters (Pro) by SlMYC2 and MED25. Effector plasmids containing the full-length coding sequences of *SlMYC2* and *MED25* were co-transfected into tobacco protoplasts with reporter plasmids containing *SlMYB12pro*. Data are shown as means ± SD (n = 3). Values marked with different letters indicate statistically significant differences as analyzed by a one-way ANOVA test from three independent biological replicate experiments. (j) ChIP-qPCR analysis of H3K9Ac and H3K27Ac levels at the *SlMYB12* promoter in WT and *slmyc2* fruits at the Br stage. Data are shown as means ± SD with three biological replicates. (k) Tentative model of the role of SlMYC2 in tomato fruit flavonoid accumulation. SlMYC2-MED25 complex activates the transcription of *SlMYB12* by directly binding to its promoter, thereby positively regulating the expression of downstream flavonoid biosynthesis genes *FLS*, *F3'H*, *F3H*, and *CHS*. This mechanism facilitates the accumulation of flavonoids in tomato fruits.
