# Supplementary material for: SlMYC2‐SlMYB12 module orchestrates a hierarchical transcriptional cascade that regulates fruit flavonoid metabolism in tomato
Source: Plant Biotechnol J. 2024 Nov 7;23(2):477–9. doi: 10.1111/pbi.14510 (PMC11772319; doi:10.1111/pbi.14510)
Supplement: Supplementary file 1 — Figure S1 The expression levels of SlMYC2 vary across different tissues and subcellular localization analysis of SlMYC2. Figure S2 The expression levels of CHS1, CHS2, HCT, CH3, F3H, F3′H, FLS and MYB12 were obtained from the qPCR data. Figure S3 RNA‐seq analysis of WT and SlMYC2‐KO fruits. Figure S4 SlMYC2 does not affect tomato fruit ripening process and carotenoid accumulation. Figure S5 Verification of interaction between SlMYC2 and MED25. Table S1 Putative transcriptional targets of the SlMYC2–SlMED25 complex by combining RNA‐seq and ChIP‐seq data. Data Set S1 Differentially expressed genes (DEGs) between slmyc2 and WT fruits. Data Set S2 Gene Expression (TPM) in slmyc2 and WT Fruits. Data Set S3 The gene locus numbers in the venn diagram of Figure S3a. Data Set S4 The kyoto encyclopedia of genes and genomes (KEGG) analysis of DEGs between slmyc2 and WT fruits. Data Set S5 Gene expression levels (TPMs) in the heat maps of Figure 1b and Figure S4e. Data Set S6 List of primers used in this study. Data Set S7 Flavonoid content and reference standard detection. Data Set S8 Carotenoid content and reference standard detection. Data Set S9 Statistical analysis. [file PBI-23-477-s001.zip › pbi14510-sup-0004-supinfo.docx]

**Supplementary Materials and Methods**

**Construction of plasmids and plant transformation**

Two guide RNA sequences targeting *SlMYC2* were designed using the CRISPR-P web application tool (<http://crispr.hzau.edu.cn/cgi-bin/CRISPR2/CRISPR>) and cloned into the pFASTCas9/ccdB binary vector to generate a CRISPR/Cas9 genome-editing plasmid vector for *SlMYC2*. Subsequently, the constructed vectors were introduced into the Micro-Tom tomato cultivar via *Agrobacterium*-mediated transformation.

**Plant growth conditions**

Tomato plants (*Solanum lycopersicum* L. Micro-Tom) were cultivated in a greenhouse under controlled conditions, including a 14-h day/10-h night photoperiod, temperatures of 25°C/20°C (day/night), 80% relative humidity, and a light intensity of 250 μmol m^–2^ s^–1^. The fruits were tagged at the breaker (Br) stage. Subsequently, the fruits were harvested at the Br+7 stage for flavonoid content measurements.

**RNA-Seq analysis**

RNA-Seq analysis was conducted by extracting total RNA from both the WT and *slmyc2-2* lines, each with three biological replicates. The extracted RNA was used for library construction and high-throughput sequencing at GenePlus (Shenzhen, China) using the Illumina Platform. The paired reads were mapped to the tomato reference genome SL4.0 with the ITAG4.0 annotation, and the transcripts were constructed using the HISAT2 tool (Kim *et al.,* 2015). Differentially expressed genes were identified using a threshold of *P* < 0.05 and absolute log2 ratio > 2. Kyoto encyclopedia of genes and genomes (KEGG) enrichment analysis was performed using the ClusterProfiler package in R (Supplemental Dataset S4) (Yu *et al.,* 2012). The gene expression levels of flavonoid and carotenoid biosynthesis in the heat maps of Figure 1b and Figure S4e are listed in Supplemental Dataset S5.

**RNA extraction and RT-qPCR analysis**

For RNA extraction and RT-qPCR analysis, total RNA was isolated from fruit pericarps using Plant RNA Purification Reagent (12322-012; Invitrogen, Waltham, MA, USA) according to the manufacturer’s instructions. First-strand complementary DNA was synthesized from mRNA using an Omniscript Reverse Transcription kit (RR047; Takara, Beijing, China) as per the manufacturer’s instructions. Gene expression was quantified by qPCR according to the method described by Deng *et al.* (2022), using SYBR Green Supermix (BG0014; Bioground, Chongqing, China) on a CFX384 real-time PCR machine (Bio-Rad, CA, USA). All the primers used are listed in Supplemental Dataset S6.

**Extraction and determination of total flavonoids**

Fresh tomato pericarp tissue at the Br+7 stage was used for flavonoid analysis. The metabolic compounds were measured according to the method described by Yuan et al. (2022). Briefly, 50 mg of dried powder was extracted in 5 mL of 80% (v/v) methanol for 16 h at 4°C with agitation. The extract was centrifuged at 3000 × *g* for 15 min at 4°C, and the residue was re-extracted with 5 mL of 80% (v/v) methanol at 4°C for 2 h. After centrifuging, supernatants were collected and combined. A 20 μL aliquot of the supernatant was mixed with 30 μL of 5% NaNO_2_ (w/v) at 25°C for 6 min. Subsequently, 50 μL of 10% AlCl3 (w/v) was added and mixed for another 6 min. Finally, 100 μL of 10% NaOH (w/v) was added. After a 15-min reaction, the flavonoid content was analyzed using a Sciex QTRAP 6500 (SCIEX, Shanghai, China) system. The compounds were quantified using standards purchased from Sigma-Aldrich (https://www.sigmaaldrich.com/). The flavonoid levels and standard detection results are listed in Supplemental Dataset S7.

**Extraction and determination of carotenoids**

At the Br+7 stage, 15 fruits each from the WT and *slmyc2* lines were harvested. Each sample was rapidly frozen in liquid nitrogen, ground into a powder, and freeze-dried for carotenoid extraction. A 100 μL aliquot of the extraction solution (hexane:acetone:ethanol, 1:1:2, v/v) was added to 0.1 g of the freeze-dried sample, followed by sonication at 25°C for 20 min and centrifuging at 8000 × *g* for 5 min at 4°C. The supernatant was collected and the extraction process was repeated twice. The combined supernatants were concentrated for approximately 15 min using a high-speed concentrator, reconstituted with 1 mL methanol, and stored in amber vials for subsequent liquid chromatography-mass spectrometry (LC-MS) analysis using a Sciex QTRAP 6500 system (SCIEX, Shanghai, China), as described by Deng *et al.* (2024)*.* The compounds were quantified using standards purchased from Sigma-Aldrich (https://www.sigmaaldrich.com/). Carotenoid levels and standard detection results are listed in Supplemental Dataset S8.

**Electrophoretic mobility shift assay**

The full-length coding sequence of *SlMYC2* was cloned into the pGEX-4T-1 vector and expressed as a GST-SlMYC2 fusion protein in BL21 Competent Cells. The fusion protein was purified using Glutathione Sepharose 4B, according to the manufacturer’s instructions (GE17-0756-01; GE Healthcare, Chicago, IL, USA). A probe containing the SlMYC2 binding site (CACAGG) from the *SlMYB12* promoter was biotin-labeled using a 3-biotin labeling kit (89818; Thermo Fisher, Waltham, MA, USA).

Unlabeled probes served as competitors, and probes mutated with CACAGG changed to AAAAAA were used as non-competitors. Electrophoretic mobility shift assay (EMSA) was performed using an EMSA/Gel-Shift Kit (GS002; Beyotime, Beijing, China) according to the manufacturer’s instructions. The reaction products were subjected to 5% (w/v) native polyacrylamide gel electrophoresis and visualized using SYBR Green and SYPRO Ruby stains (E33075; Thermo Fisher, Waltham, MA, USA) for fluorescence detection.

**Transient expression assays**

The transient expression assays were performed as described by Deng *et al.* (2022). The coding sequences of *SlMYC2* and *MED25* were cloned into the pGreenII 62sk vector as effectors. The promoter of *SlMYB12* was cloned into the pGreenII 0800-LUC vector as the reporter. The effector and reporter constructs were co-transfected into mesophyll protoplasts isolated from *N. benthamiana* leaves using polyethylene glycol-mediated transfection, as described by Deng *et al.* (2022). Approximately 10 to 16 hours post-transfection, LUC and REN activities were measured using a dual LUC assay kit (E1910; Promega, Madison, WI, USA) according to the manufacturer’s instructions.

**DNA pull-down**

The DNA pull-down assay was performed as described by Deng *et al.* (2022). Recombinant MBP-MED25 and MBP-SlMYC2 proteins were produced from the BL21 codon plus *Escherichia coli* cells and purified. The promoter fragment of SlMYB12 was amplified by PCR using the 5′-biotin-labeled primers. For the assay, 10 µg of MBP-MED25 was incubated with the biotin-labeled DNA together with 10 µg of MBP-SlMYC2 protein, whereas control samples were incubated overnight without MBP-SlMYC2 protein in HKMG buffer (10 mM HEPES, pH 7.9, 100 mM KCl, 5 mM MgCl2, 10% [v/v] glycerol, 1 mM DTT, and 0.5% [v/v] NP-40) containing protease and phosphatase inhibitors.

**Statistical analysis**

Statistical analyses were performed using GraphPad Prism 8 software. For the transient expression assays, one-way analysis of variance (ANOVA) was used to determine significant differences and statistical significance was set at *p* < 0.05. Remaining data were analyzed using an unpaired Student’s *t*-test, with statistical significance set at *p* < 0.05. All the statistical analysis results are presented in Supplemental Dataset S9.

**Accession numbers**

The sequence data from the article can be found in the Tomato Genome Protein Sequences (ITAG release 4.0) database. The specific accession numbers for the genes discussed are as follows: *SlMYC2* (*Solyc08g076930*), *SlMED25* (*Solyc12g070100*), *SlMYB12* (*Solyc02g077790*), *PAL* (*Solyc03g071870*), *C4H* (*Solyc05g047530*), *CHS1* (*Solyc09g091510*), *CHS2* (*Solyc05g053550*), *HCT* (*Solyc03g117600*), *4CL* (*Solyc03g117870*), *HQT* (*Solyc07g005760*), *CHI1* (*Solyc05g010320*), *CHI1-like* (*Solyc05g052240*), *F3H* (*Solyc02g083860*), *F3’H* (*Solyc03g115220*), *FLS* (*Solyc11g013110*) and 3*GT* (*Solyc10g083440*).

**Reference**

Deng, H., Chen, Y., Liu, Z., Liu, Z., Shu, P., Wang, R., Hao, Y., *et al.* (2022) SlERF.F12 modulates the transition to ripening in tomato fruit by recruiting the co-repressor TOPLESS and histone deacetylases to repress key ripening genes. Plant Cell. **34**, 1250-1272.

Deng, H., Pei, Y., Xu, X., Du, X., Xue, Q., Gao, Z., Shu P., *et al.* (2024) Ethylene-MPK8-ERF.C1-PR module confers resistance against Botrytis cinerea in tomato fruit without compromising ripening. *New Phytologist.* **242,** 592-609.

Kim, D., Langmead, B., Salzberg, S.L. (2015) HISAT: a fast spliced aligner with low memory requirements. Nat Methods. **12**(4), 357–360.

Yu, G., Wang, L.G., Han, Y., He, Q.Y. (2012) clusterProfiler: an R package for comparing biological themes among gene clusters. Omics. **16**(5), 284–287.

Yuan, Y., Ren, S., Liu, X., Su, L., Wu, Y., Zhang, W., Li, Y., *et al.* (2022) SlWRKY35 positively regulates carotenoid biosynthesis by activating the MEP pathway in tomato fruit. New Phytol. **234**(1), 164–178.

Zhang, D., Tan, W., Yang, F., Han, Q., Deng, X., Guo, H., Liu, B., *et al.* (2021) A BIN2-GLK1 Signaling Module Integrates Brassinosteroid and Light Signaling to Repress Chloroplast Development in the Dark. *Dev Cell.* **56**, 310-324.
