# Supplementary material for: SlMYC2‐SlMYB12 module orchestrates a hierarchical transcriptional cascade that regulates fruit flavonoid metabolism in tomato
Source: Plant Biotechnol J. 2024 Nov 7;23(2):477–9. doi: 10.1111/pbi.14510 (PMC11772319; doi:10.1111/pbi.14510)
Supplement: Supplementary file 1 — Figure S1 The expression levels of SlMYC2 vary across different tissues and subcellular localization analysis of SlMYC2. Figure S2 The expression levels of CHS1, CHS2, HCT, CH3, F3H, F3′H, FLS and MYB12 were obtained from the qPCR data. Figure S3 RNA‐seq analysis of WT and SlMYC2‐KO fruits. Figure S4 SlMYC2 does not affect tomato fruit ripening process and carotenoid accumulation. Figure S5 Verification of interaction between SlMYC2 and MED25. Table S1 Putative transcriptional targets of the SlMYC2–SlMED25 complex by combining RNA‐seq and ChIP‐seq data. Data Set S1 Differentially expressed genes (DEGs) between slmyc2 and WT fruits. Data Set S2 Gene Expression (TPM) in slmyc2 and WT Fruits. Data Set S3 The gene locus numbers in the venn diagram of Figure S3a. Data Set S4 The kyoto encyclopedia of genes and genomes (KEGG) analysis of DEGs between slmyc2 and WT fruits. Data Set S5 Gene expression levels (TPMs) in the heat maps of Figure 1b and Figure S4e. Data Set S6 List of primers used in this study. Data Set S7 Flavonoid content and reference standard detection. Data Set S8 Carotenoid content and reference standard detection. Data Set S9 Statistical analysis. [file PBI-23-477-s001.zip › pbi14510-sup-0005-supinfo.docx]

**Table S1.** Putative transcriptional targets of the SlMYC2–SlMED25 complex by combining RNA-seq and ChIP-seq data.

| Locus | Note | Family | Class | Log2(Ma/WT) | Log2(*myc2*/WT) |
| --- | --- | --- | --- | --- | --- |
| Solyc01g079620 | Colorless fruit epidermis | MYB | TF | -1.92 | -1.01 |
| Solyc03g098320 | Protein REVEILLE 1 (AHRD V3.3 *** A0A2G2XWA2_CAPAN) | MYB-related | TF | -2.64 | -1.43 |
| Solyc07g006630 | Zinc finger protein CONSTANS-like 5-like (AHRD V3.3 *** A0A2K3N2C5_TRIPR) | C2C2-CO-like | TF | -3.58 | -1.19 |
| Solyc05g011890 | Sulfotransferase (AHRD V3.3 *** A0A2G3B6Y2_CAPCH) | SG | SG | -4.69 | -1.12 |
| Solyc07g062670 | Unknown protein | SG | SG | -9.12 | -2.00 |
| Solyc05g005170 | Pectin lyase-like superfamily protein (AHRD V3.3 *** O22699_ARATH) | SG | SG | -6.60 | -6.70 |
| Solyc04g078460 | Isoaspartyl peptidase/L-asparaginase (AHRD V3.3 *** A0A1U8GBV5_CAPAN) | SG | SG | -2.66 | -1.45 |
| Solyc09g089620 | AT-hook motif nuclear-localized protein (AHRD V3.3 *** A0A2G3BF72_CAPCH) | SG | SG | -7.14 | -0.91 |
| Solyc07g053640 | Arabinogalactan-protein (AHRD V3.3 *-* Q41256_NICAL) | SG | SG | -6.39 | -1.05 |
| Solyc10g083690 | Cytochrome P450 (AHRD V3.3 *** Q8H0I8_PETHY) | SG | SG | -1.54 | -0.96 |
| Solyc02g076680 | DUF688 domain-containing protein (AHRD V3.3 *** A0A1Q3BIV2_CEPFO) | SG | SG | -6.09 | -0.93 |
| Solyc10g008660 | Anthocyanidin 3-O-glucoside-6''-O-malonyltransferase (AHRD V3.3 *-* Q6WB13_CHRMO) | SG | SG | -5.62 | -0.96 |
| Solyc07g054760 | Wound-responsive family protein (AHRD V3.3 *** A0A2K3L288_TRIPR) | SG | SG | -2.01 | -1.00 |
| Solyc06g066520 | Avr9/Cf-9 rapidly elicited protein 146 (AHRD V3.3 *** Q9FQZ6_TOBAC) | SG | SG | -2.25 | -1.59 |
| Solyc02g065240 | Methyl esterase 1 (AHRD V3.3 *** A0A2U1LMY0_ARTAN) | SG | SG | -9.65 | -1.35 |
| Solyc01g102610 | Ferric reduction oxidase 6 (AHRD V3.3 *** A0A2G3BUN6_CAPCH) | SG | SG | -2.57 | -1.40 |
| Solyc03g097560 | Bidirectional sugar transporter SWEET (AHRD V3.3 *** A0A142DVI0_SOLTU) | SG | SG | -5.29 | -1.45 |
| Solyc06g076160 | Cytochrome P450 (AHRD V3.3 *** A0A200RCX6_9MAGN) | SG | SG | -9.05 | -1.46 |
